# Identification of biomarkers for the diagnosis of chronic kidney disease (CKD) with non-alcoholic fatty liver disease (NAFLD) by bioinformatics analysis and machine learning

**DOI:** 10.3389/fendo.2023.1125829

**Published:** 2023-02-27

**Authors:** Yang Cao, Yiwei Du, Weili Jia, Jian Ding, Juzheng Yuan, Hong Zhang, Xuan Zhang, Kaishan Tao, Zhaoxu Yang

**Affiliations:** ^1^ Department of Hepatobiliary Surgery, Xijing Hospital, The Fourth Military Medical University, Xi’an, China; ^2^ Department of Nephrology, Tangdu Hospital, The Fourth Military Medical University, Xi’an, China; ^3^ Department of General Surgery, Xijing Hospital, The Fourth Military Medical University, Xi’an, China

**Keywords:** hub genes, chronic kidney disease, non-alcoholic fatty liver disease, immune, inflammation

## Abstract

**Background:**

Chronic kidney disease (CKD) and non-alcoholic fatty liver disease (NAFLD) are closely related to immune and inflammatory pathways. This study aimed to explore the diagnostic markers for CKD patients with NAFLD.

**Methods:**

CKD and NAFLD microarray data sets were screened from the GEO database and analyzed the differentially expressed genes (DEGs) in GSE10495 of CKD date set. Weighted Gene Co-Expression Network Analysis (WGCNA) method was used to construct gene coexpression networks and identify functional modules of NAFLD in GSE89632 date set. Then obtaining NAFLD-related share genes by intersecting DEGs of CKD and modular genes of NAFLD. Then functional enrichment analysis of NAFLD-related share genes was performed. The NAFLD-related hub genes come from intersection of cytoscape software and machine learning. ROC curves were used to examine the diagnostic value of NAFLD related hub genes in the CKD data sets and GSE89632 date set of NAFLD. CIBERSORTx was also used to explore the immune landscape in GSE104954, and the correlation between immune infiltration and hub genes expression was investigated.

**Results:**

A total of 45 NAFLD-related share genes were obtained, and 4 were NAFLD-related hub genes. Enrichment analysis showed that the NAFLD-related share genes were significantly enriched in immune-related pathways, programmed cell death, and inflammatory response. ROC curve confirmed 4 NAFLD-related hub genes in CKD training set GSE104954 and other validation sets. Then they were used as diagnostic markers for CKD. Interestingly, these 4 diagnostic markers of CKD also showed good diagnostic value in the NAFLD date set GSE89632, so these genes may be important targets of NAFLD in the development of CKD. The expression levels of the 4 diagnostic markers for CKD were significantly correlated with the infiltration of immune cells.

**Conclusion:**

4 NAFLD-related genes (DUSP1, NR4A1, FOSB, ZFP36) were identified as diagnostic markers in CKD patients with NAFLD. Our study may provide diagnostic markers and therapeutic targets for CKD patients with NAFLD.

## 1 Introduction

Chronic kidney disease (CKD) is defined as structural or functional abnormalities of the kidney caused by various causes for ≥ 3 months (1). Epidemiological studies show that there are approximately 434.3 million people with CKD in Asia, most of whom come from developing countries like China and India (2). The effective control of chronic kidney disease is a huge public health challenge worldwide (3).

Previous studies have suggested that acute kidney injury, hypertension, and diabetes are risk factors for CKD (4). Recently, accumulating evidence indicates that Non-alcoholic fatty liver disease (NAFLD) may be associated with the development of CKD (5–8).

NAFLD is a heterogeneous disease in which the vast majority are non-alcoholic fatty liver (NAFL) and less than 20% are non-alcoholic steatohepatitis (NASH). Nash has typical characteristics which include inflammation, hepatocyte ballooning, and hepatic injury with or without fibrosis (9, 10). NAFLD is often associated with a variety of metabolic diseases, including hypertension, diabetes, insulin resistance, etc, which are risk factors for CKD. However, the degree of fibrosis in NAFLD was independently associated with CKD progression even when confounding factors such as metabolic diseases were excluded (11–15). Excess fat may association with CKD progression in NAFLD patients by inducing lipotoxicity, inflammation, oxidative stress and fibrosis through pro-inflammatory adipokines and lipocalin (16, 17).

Despite growing evidence of the strong association between NAFLD and CKD, the key molecules and potential mechanisms involved remain unclear. Here, bioinformatics and machine learning were attempted to discover the diagnostic markers and related signaling pathways of CKD in the context of NAFLD, which were hoped to provide a basis for the clinical treatment of CKD patients with NAFLD.

## 2 Materials and methods

### 2.1 Data acquisition and preliminary processing

**Figure 1:**
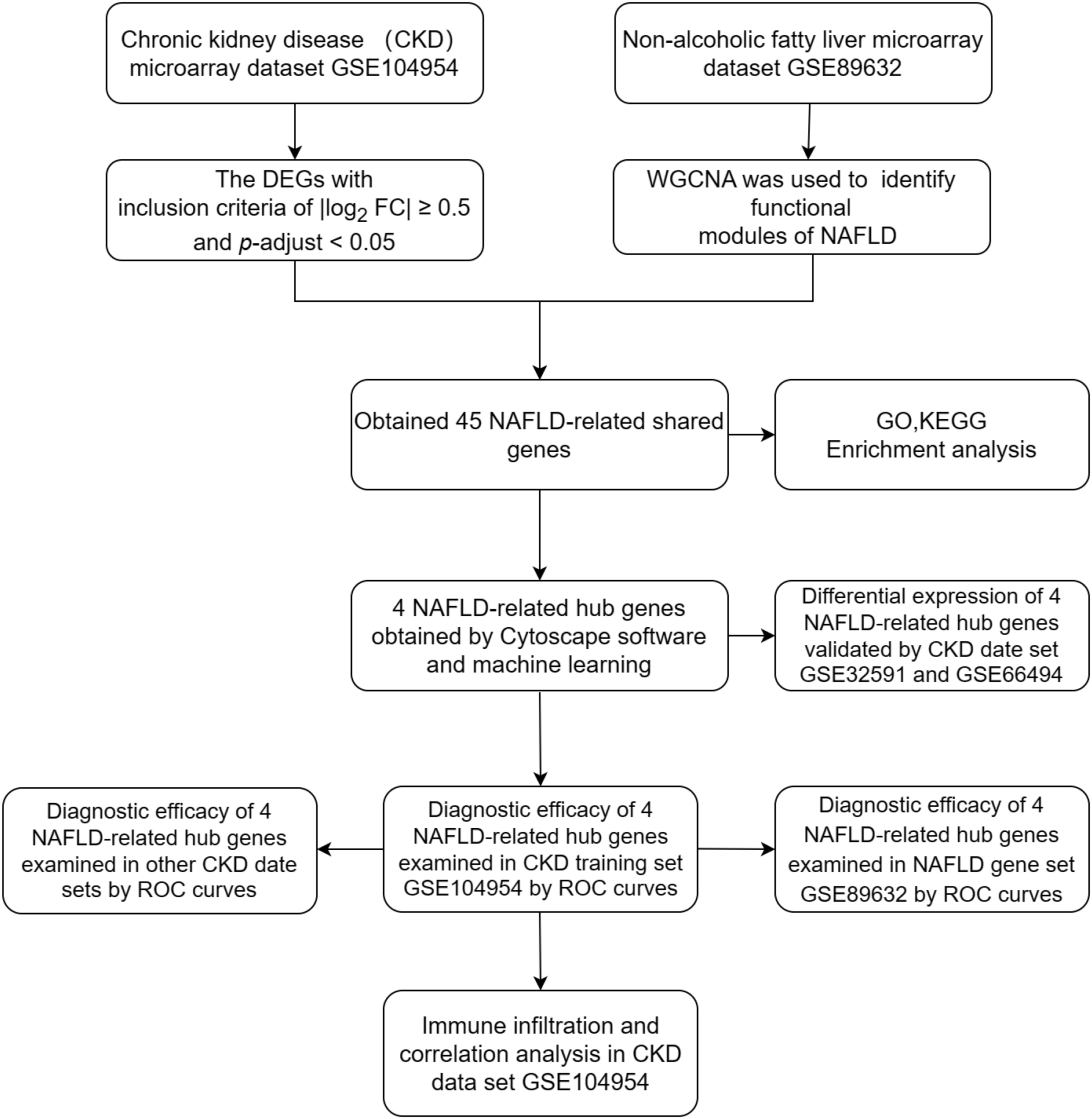
Roadmap of the main research ideas in this article.

Four data sets [GSE104954, GSE104948 (18), GSE32591 (19),GSE66494 (20)] containing gene expression profiles for Chronic kidney disease (CKD) samples and one date set [GSE89632 (21)] of non-alcoholic fatty liver disease (NAFLD) were downloaded from the GEO database (https://www.ncbi.nlm.nih.gov/geo/).Details for the data sets were provided in **Table 1**. The principal search flow of the article was illustrated in **Figure 1**.

**Table 1:** Details regarding the 5 data sets, type of samples, test platforms, numbers of samples, samples application types and source documentation.

Data sets	Platforms	Type of Samples	Control sample size	CKD or NAFLD sample size	Applications	References (PMID)
GSE104954 or GSE104948	GPL22945 GPL24120	CKD	21	169	Discovery of DEGs	29724730
GSE89632 GSE32951	GPL14951 GPL14663	NAFLD	24 29	39 64	Discovery of Modular genes Validation of hub genes	25581263 22723521
GSE66494	GPL6480	CKD	8	53	Validation of hub genes	26317775

### 2.2 Weighted gene co-expression network analysis and module gene selection in NAFLD patients

The WGCNA method was used to construct gene coexpression networks and identify functional modules. First, the median absolute deviation (MAD) of each gene was determined, and genes with MAD values in the bottom 50% were removed. Second, ineligible genes and samples were removed with the goodSamplesGenes function, and a scale-free coexpression network was built. Third, an appropriate “soft” threshold power (β) was determined to calculate intergenic adjacency; then, the adjacency values were converted into a topological overlap matrix (TOM), and gene proportions and phase dissimilarities are determined. Fourth, modules were detected using hierarchical clustering and dynamic tree cutting functions. Finally, gene significance (GS) and module membership (MM) correlations were calculated, and the corresponding module gene information was extracted for further analysis.

### 2.3 Identification of differentially expressed genes between CKD samples and controls

The DEGs were found in GSE104954 data set using the “limma” R package with inclusion criteria of |log_2_ FC| ≥ 0.5 and *p*-adjust < 0.05. DEGs were shown by volcano and expression levels of the 50 most significantly expressed genes were displayed by heatmaps, respectively.

### 2.4 Acquisition of NAFLD-related shared genes

Intersection of DEGs in GSE104954 and WGCNA module genes in GSE89632 were defined as NAFLD-related shared genes, represented by a Venn schema by the online website jvenn (http://jvenn.toulouse.inra.fr/app/example.html).

### 2.5 Enrichment analysis

For enrichment analysis of NAFLD-related shared genes, Gene Ontology (GO) annotations of genes from the R package org.Hs.eg.db based on the R package “clusterProfiler”, and the minimum number of genes per gene set was 5 and the maximum was 5000. Gene annotations for Kyoto Encyclopedia of Genes and Genomes (KEGG) pathways were operated through KOBAS-i online tool (http://kobas.cbi.pku.edu.cn/) (22), and a false discovery rate <0.05 was considered statistically significant.

### 2.6 Establishment of protein-protein interaction network and identification of NAFLD-related hub genes by cytoscape software and machine learning

The NAFLD-related shared genes were uploaded to the STRING database (http://string-db.org/) to construct the PPI network with a PPI score threshold (medium confidence≥0.700). Hub genes were screened by the Cytoscape (Version 3.9.1) plug-in APP MCODE (Version 2.0.0). At the same time, the machine learning methods random forest (RF) were used to screen for hub genes. The MeanDecreaseGini (MDG) was used to measure the importance of genes by the RF algorithm with “randomForest” package, and hub genes were defined as MDG greater than 1.5. The final NAFLD-related hub genes were obtained by the intersection of the results of cytoscape software and machine learning.

### 2.7 Verification of NAFLD-related hub genes expression in the CKD data sets

Data set GSE32591 and GSE66494 were used to identify the expression level of the hub genes. GSE 32591 is composed of 29 control samples and 64 samples with lupus nephritis. GSE66494 contains 53 biopsy samples, including 8 control samples and 45 CKD samples.

### 2.8 Construction of receiver-operating characteristic curves to assess diagnostic efficacy

ROC curves were constructed by “pROC” package in Xiantao Academic (https://www.xiantao.love/products) to evaluate the diagnostic value of NAFLD-related hub genes in the CKD training set GSE104954 and other CKD validation date sets (GSE32591,GSE66494 and GSE104948). Further the diagnostic value of hub gene in the NAFLD data set GSE89632 was also evaluated.

### 2.9 Immune infiltration analysis and correlation analysis

The composition and abundance of 22 types immune cells can be estimated from the CKD and control samples transcriptome in GSE104954 date set by CIBERSORTx (https://cibersortx.stanford.edu/). The correlations of NAFLD-related hub genes expression with immune cell infiltrations were investigated in Xiantao Academic, as were their respective correlations.

### 2.10 Statistical analysis

All statistical analyses of bioinformatics studies in this study were conducted using R software. The differences between the groups were tested using a nonparametric Wilcoxon signed-rank test. Correlation analysis was performed using Spearman’s correlation. In comparison, p < 0.05 was considered statistically significant (*p < 0.05, **p < 0.01, ***p<0.001, ****p<0.0001).

## 3 Results

### 3.1 Key module genes in NAFLD samples were identified by WGCNA

**Figure 2:**
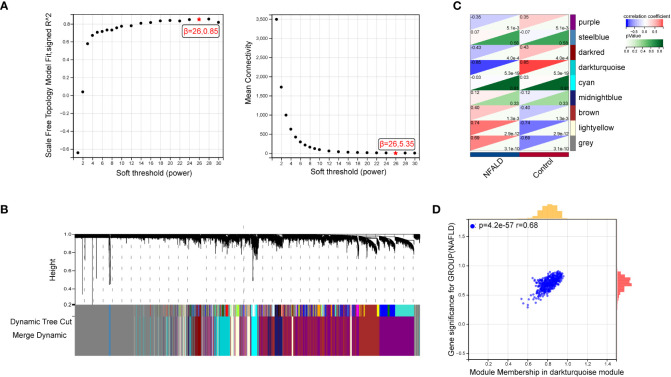
Identification of module genes *via* WGCNA in NAFLD date set GSE89632. **(A)** β = 26 is selected as the “soft” threshold with the combined analysis of scale independence and average connectivity. **(B)** Gene coexpression modules represented by different colors under the gene tree. **(C)** Correlation plot between module membership and gene significance of genes included in the darkturquoise module. **(D)** Heatmap of the association between the darkturquoise modules and NAFLD samples. NAFLD, Non-alcoholic fatty liver disease; WGCNA, weighted gene co-expression network analysis.

GSE89632 is a representative dataset for investigation of NAFLD and we used it to obtain the most relevant modular genes for NAFLD (23–25).First, WGCNA was used for the identification of the most relevant modular genes for NAFLD.β = 26 (scale-free R^2^ = 0.85) was selected as the “soft” threshold based on the scale independence and average connectivity (**Figure 2A**). Then, different colors are chosen to represent 9 gene co-expression modules (GCMs), which are presented in **Figure 2B**. The correlation between NAFLD samples and GCMs is shown in **Figure 2C**, and the darkturquoise module (412 genes) which was regarded as critical modules demonstrated the highest correlation with NAFLD samples (correlation coefficient = -0.85, *p* = 5.3e-19). In addition, a significant positive correlation was observed between module membership and gene significance in darkturquoise modules for NAFLD samples (r = 0.68, *p*=4.2e-57), as shown in **Figure 2D**.

### 3.2 Identification of NAFLD-related shared genes between CKD and NAFLD

**Figure 3:**
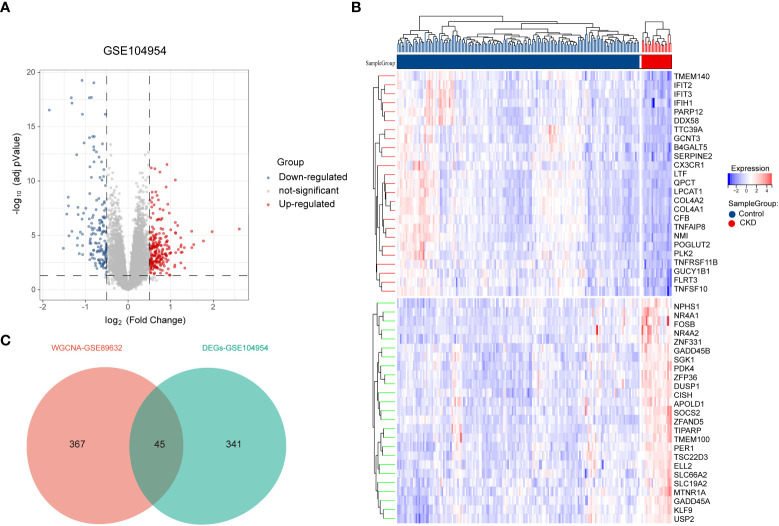
Screening of differentially expressed genes (DEGs) and NAFLD-related share genes. **(A)** The volcano plot of DEGs in CKD date set GSE104954.**(B)** Heat map of the 50 most significantly differentially expressed genes, where red and green indicate the most significantly up-regulated and down-regulated differentially expressed genes in the CKD samples, respectively. **(C)** NAFLD related shared genes were obtained by intersection of NAFLD module genes obtained by WGCNA in date set GSE89632 and DEGs from CKD in date set GSE104954. NAFLD, Non-alcoholic fatty liver disease; CKD, chronic kidney diseases.

Next, 386 DEGs were found in GSE104954 data set, of which 227 were up-regulated, and 159 of these genes were down-regulated. **Figure 3A** shows the DEGs by the volcano diagram. The heatmap of the top 50 most significant DEGs in the data set is plotted in **Figure 3B**. Then, 386 DEGs and 412 module genes were intersected, and 45 NAFLD-related shared genes were subsequently obtained, as presented in the Venn diagram in **Figure 3C** (Detailed results were provided in **Supplementary Materials S1**).

### 3.3 Enrichment analyses of 45 NAFLD-related shared genes

**Figure 4:**
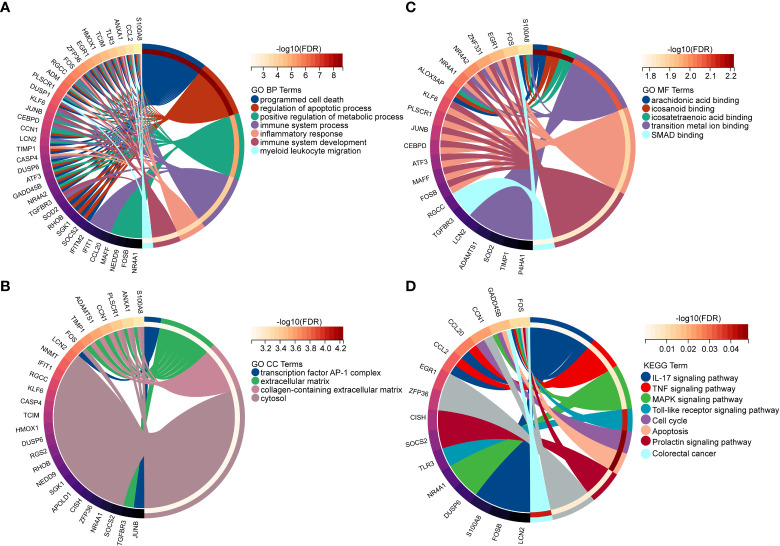
Enriched items in GO and KEGG analyses of 45 NAFLD share genes. **(A)** Enriched items in the GO BP analysis. **(B)** Enriched items in the GO CC analysis. **(C)** Enriched items in the GO MF analysis. **(D)** Enriched items in the KEGG pathway analysis. NAFLD, Non-alcoholic fatty liver disease; GO, gene ontology; BP, biological process; CC, cellular component; MF, molecular function; KEGG, Kyoto Encyclopedia of Genes and Genomes; NAFLD, Non-alcoholic fatty liver disease.

In order to explore the biological functions and pathways of NAFLD-related shared genes in the development of CKD, GO and KEGG enrichment analyses were performed for 45 shared genes. A total of 563 significantly related biological processes and 23 KEGG signaling pathways were obtained (Detailed results were provided in **Supplementary Materials S2**). GO analysis of shared genes was performed to reveal their biological functions (**Figures 4A–C**). As we have seen, in the GO category, most of the share genes mostly involved in the “programmed cell death”, “inflammatory response”, “positive regulation of metabolic process”, and “immune system process”(BP); “Extracellular matrix”, “collagen-containing extracellular matrix” (CC); “DNA-binding transcription activator activity, RNA polymerase II-specific”, “DNA-binding transcription factor activity”, etc (MF). The results of KEGG pathway enrichment showed that the most involved pathways were the IL-17 signaling pathway, TNF signaling pathway, MAPK signaling pathway, Apoptosis, Toll-like receptor signaling pathway, and so on, which are closely related to the immune response and inflammation (**Figure 4D**).

### 3.4 Identification of NAFLD-related hub genes *via* cytoscape software and machine learning and their differential expression was validated

**Figure 5:**
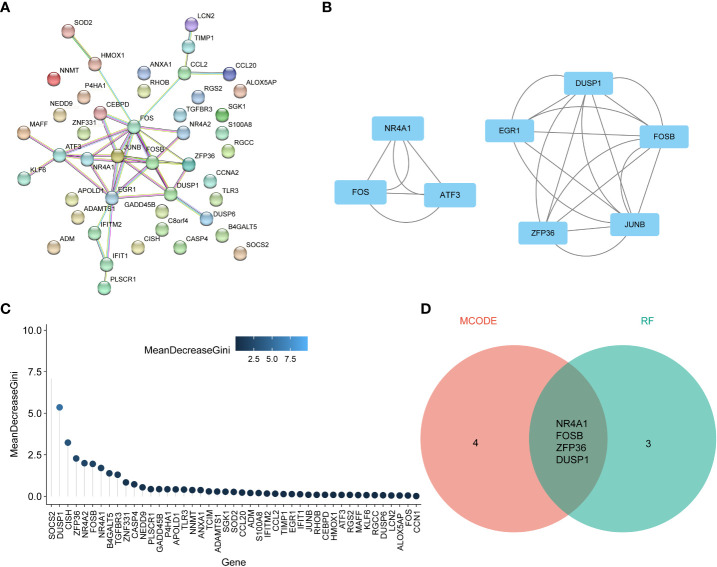
Identification of NAFLD-related hub genes by the intersection of cytoscape software and machine learning. **(A)** Protein-protein interaction (PPI) network of 45 NAFLD-related share genes. **(B)** The 8 genes in two clusters with the most significant associations were accessed using the MCODE plug-in. **(C)** Random Forest analysis for NAFLD-related hub DEGs. **(D)** Venn diagram demonstrates the final NAFLD-related hub genes obtained by the intersection of cytoscape software and machine learning. DEGs, differentially expressed genes; NAFLD, Non-alcoholic fatty liver disease; MCODE, molecular complex detection.

**Figure 6:**
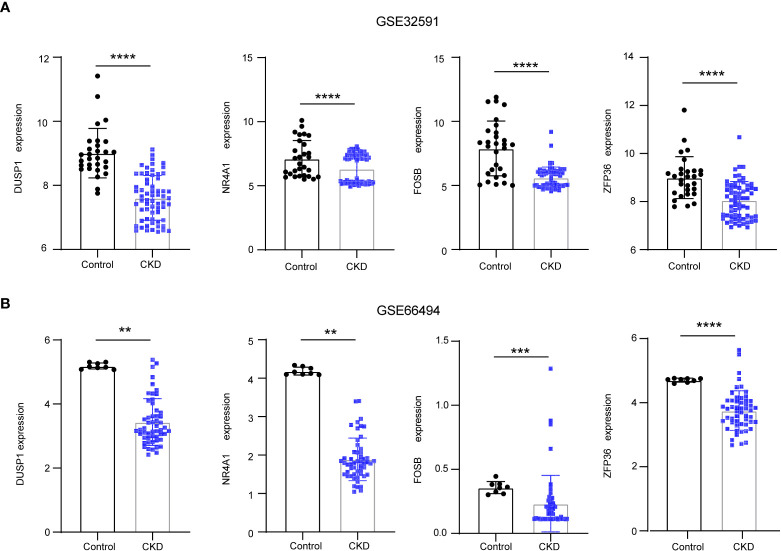
The downregulation of 4 NAFLD-related hub genes was verified by two CKD data set. **(A)** Expression of NAFLD-related hub genes in the GSE32591 date set. **(B)** Expression of NAFLD-related hub genes in the GSE66494 date set. NAFLD, Non-alcoholic fatty liver disease; CKD, chronic kidney diseases.

To reveal the interaction of each protein, the PPI network of the shared genes were built according to the STRING database. There were 38 edges and 45 nodes in **Figure 5A**, followed by analysis using Cytoscape software. MCODE plugin was used to discover the important modules in the PPI network and the results showed that 8 hub genes in two clusters were tightly connected as the important modules in **Figure 5B**. On the other hand,7 hub genes with MeanDecreaseGini> 1.5 were determined by the random forest algorithm in **Figure 5C**. A Venn diagram in **Figure 5D** showed the intersection of 4 hub genes (DUSP1, FOSB, NR4A1, ZFP36), which were used as NAFLD-related hub genes. Moreover, in the other two CKD datasets (GSE32591, GSE66494), the 4 NAFLD-related genes were significantly down-regulated (**Figures 6A, B**), which was consistent with the change in GSE104954 (**Supplementary Materials S3**).

### 3.5 The ROC curve was used to evaluate diagnostic efficacy in CKD and NAFLD

**Figure 7:**
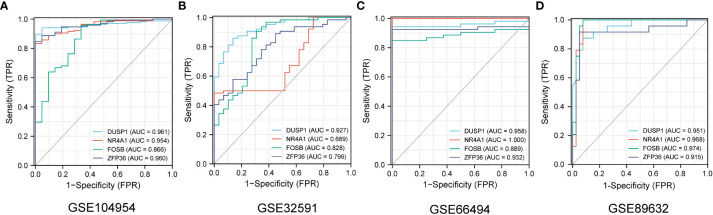
The diagnostic efficacy of 4 NAFLD-related hub genes was verified by ROC curve in CKD and NAFLD data sets. **(A–D)** The ROC curve of 4 NAFLD-related hub genes in date set GSE104954, GSE32951, GSE66494 and GSE89632.

The ROC curves of 4 NAFLD-related genes (DUSP1, FOSB, NR4A1, ZFP36) with AUCs of 0.961, 0.954, 0. 866, and 0.960 in the training set GSE104954, respectively (**Figure 7A**). Meanwhile, in the validation set GSE32591, the AUCs of these hub genes were 0.828,0.796,0.927 and 0.689, respectively, which did not distinguish whether the source of the sample was glomerular or tubulointerstitial (**Figure 7B**). At the same time, in the other validation set GSE66494, the AUCs of hub genes were 0.958,1.000,0.889, and 0.932, respectively (**Figure 7C**). Comprehensive analysis of the results of the validation and training sets showed that the 4 NAFLD-related genes can serve as effective markers for the diagnosis of CKD.

GSE104948 and GSE104954, as sister datasets, represent glomerular and tubulointerstitial transcript level information of the same cohort of samples, respectively. 4 NAFLD-associated hub genes have the same good diagnostic efficacy for CKD in GSE104948 (**Supplementary Materials S4**). Similarly, in the data set GSE32591, both tubulointerstitial and glomerular samples were sampled, and further exploration revealed that the 4 diagnostic markers showed good efficacy in different anatomical structures (**Supplementary Materials S5**). What should be noted is that these 4 CKD diagnostic markers also have good diagnostic value for NAFLD in GSE89632 date set, and the ROC curves with AUCs of 0.951,0.968,0.974, and 0.915, respectively (**Figure 7D**). This finding may suggest that four genes may play a significant role in the development of CKD patients with NAFLD.

### 3.6 Immune infiltration analysis and correlation analysis

**Figure 8:**
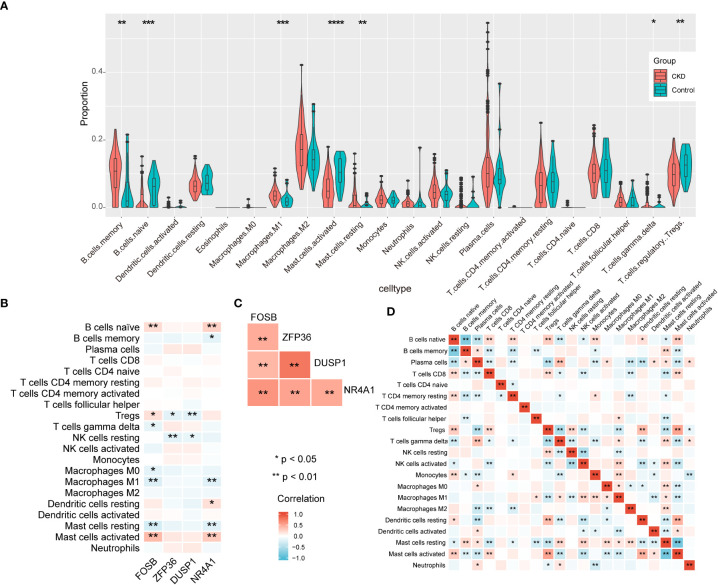
The immune landscape of CKD samples in GSE104954 and correlation analysis. **(A)** The violin theme of immune cell proportions. **(B)** The correlation between four diagnostic markers genes and immune cells. **(C)** The correlation matrix of four diagnostic markers genes. **(D)** Correlation matrix of ratios of immune cells.

According to the results of enrichment analysis, NAFLD-related shared genes may be involved in the immune-related mechanisms of CKD progression. Therefore, the correlation between the 4 diagnostic markers genes with immune cell infiltration in CKD is noteworthy for further exploration. First, CIBERSORTx was used to evaluate the proportions of 22 immune cell in GSE104954 data set (**Figure 8A**). B cells memory, Macrophages M1, Mast cells resting, T cells gamma delta were significantly upregulated in CKD samples; however, the levels of B cells naive, Treg cells, Mast cells activated were significantly decreased. Next, the correlation of the four diagnostic markers genes with CKD immune cells was explored (**Figure 8B**). The FOSB expression was positively correlated with the ratios of B cells naive, Treg cells and Mast cells activated, and negatively correlated with Mast cells resting, T cells gamma delta, M0 Macrophages and M1 Macrophages. ZFP36 and DUSP1 were negatively correlated with the ratios of Treg cells and NK cells resting.NR4A1 was positively correlated with the ratios of B cells naive, Dendritic cells resting and Mast cells activated, and negatively correlated with Mast cells resting, M1 Macrophages and B cells memory. When exploring the interrelationships between the expression of the four diagnostic markers genes, it is interesting to note that they were all positively correlated with each other (**Figure 8C**), suggesting that they may participate in a common mechanism to promote CKD development. Additionally, the interplay of immune cells was explored (**Figure 8D**). Treg cells and Mast cells activated had the strongest positive correlation with one another (r = 0.53). In contrast, resting mast cells showed the strongest negative correlation with activated mast cells (r = -0.83).

In summary, CKD samples showed significant changes in immune cell infiltration compared with controls, and the four diagnostic markers genes expression was significantly correlated with immune cell infiltration.

## 4 Discussion

NAFLD and CKD are both significant global public health burden, and there is evidence that NAFLD is independently associated with a high risk of CKD despite the exclusion of other metabolic diseases, while the underlying mechanisms are not clear (26). In our study, by obtaining DEGs and important module genes by WGCNA, 45 NAFLD-related share genes were obtained, and their enrichment analysis revealed that immune, inflammatory and programmed cell death pathways were significantly enriched. Further, 4 CKD diagnostic markers genes were obtained by cytoscape software and machine learning, which demonstrated good diagnostic value in both the training and validation sets of CKD. Interestingly, 4 CKD biomarkers also had good diagnostic performance for NAFLD in dataset GSE89632, indicating that they may be important targets for the development of CKD in NAFLD patient.

DUSP1, dual-specificity protein phosphatase 1, is a member of the dual-specific phosphatase (DUSPs) family. Mitogen-activated protein kinases (MAPKs) was closely related to inflammation and immune, and DUSP1 improves microvascular fibrosis and inflammation through dephosphorylation of MAPKs (27, 28). Overexpression of DUSP1 alleviates renal tubular injury by regulating mitophagy and interrupte Mff-related excessive mitochondrial fission. At the same time, lncRNA NR_038323 reduced the degree of renal fibrosis by targeting DUSP1, suggesting that DUSP1 is a potential therapeutic target for CKD with NAFLD (29–31). However, the main evidence come from diabetic renal disease, and whether it is applicable to other types of CKD requires further study.

FOSB is a member of the FOS family, which is part of activator protein-1 (AP-1). AP-1 is associated with immune and cancer progression. Previous studies have shown that FOSB can be used as a diagnostic marker for lgA kidney disease (32). It has also been shown that MicroRNA-27a-3p targets FOSB to regulate the level of inflammation and fibrosis in lgA nephropathy (33). Zinc finger protein 36 (ZFP36) participates in posttranscriptional regulation by targeting different mRNAs, which was closely related to inflammatory diseases and autoimmune disease (34). The dysregulated expression of ZFP36 may play an important role in the pathogenesis of inflammatory diseases including CKD. While it has been suggested that it could be used as a diagnostic marker of CKD (35, 36). Further studies are needed to identify the underlying mechanism for FOSB and ZFP36 in CKD with NAFLD.

The orphan nuclear receptor 4A1 (NR4A1), which is also known as Nur77, belongs to the nuclear receptor superfamily, and is involved in inflammation and energy metabolism pathways (37). Previous studies have shown that it can be used as a therapeutic target for chronic kidney disease, which is consistent with our study (38). There are also studies show that Yiqi Huoxue Tongluo recipe, a traditional Chinese medicine, can alleviate renal inflammation and fibrosis by increasing the expression level of NR4A1. In contrast, the loss of NA4A1 results in increased kidney ingury associated with macrophage. Interestingly, in our results, NR4A1 expression was significantly negatively correlated with proinflammatory M1 macrophage infiltration. A recent study showed that the induction of anti-inflammatory macrophages expressing NR4A1/EAR2 could suppress M1 proinflammatory responses, thereby inhibiting immune-mediated crescent glomerulonephritis (39–41). Therefore, increasing the expression level of NR4A1 may be one of the potential strategies for the treatment of CKD with NAFLD.

Dysfunction of immune cells promotes inflammation and kidney fibrosis in CKD, so the immune infiltration status of CKD samples was explored (42). Previous studies have demonstrated that macrophage polarization plays an important role in CKD development (43). In our results, M1 macrophages were significantly upregulated. However, M2 macrophages are believed to be associated with fibrosis, but not M1 macrophages. While the role of M2 macrophages in CKD is contradictory (44, 45).

Tregs cells are down-regulated in CKD, which is consistent with our results (46). There is some evidence to suggest that Tregs cells can inhibit inflammation and fibrosis in CKD (47). In turn, the CKD microenvironment changes the energetic metabolism of Tregs cells, thus inhibiting the protective effect of Tregs (48). Therefore, immune cells interact with the inflammatory microenvironment. B cells are thought to be involved in the progression of Membranous nephropathy (MN) by releasing antibodies against podocytes, so depletion therapy targeting B cells may be a potential treatment (49). However, existing data suggest that depletion of B cells does not achieve the expected effect in lgA nephropathy (50). Because of the significant heterogeneity of CKD, the potential significance of B-cell depleting therapy requires specific analysis (51). The MC-specific protease tryptase is released by mast cells, thereby activating significant fibrosis and inflammation (52). In our results, the expression of four CKD diagnostic markers was closely related to the infiltration of multiple immune cells, which also confirmed the important role of immune mechanisms in the development of inflammatory and fibrosis in CKD patients with NAFLD.

There are limitations to our study. First, CKD is an umbrella term with significant heterogeneity, and we were not able to analyze specific types of CKD; Second, we focus on the pathogenesis and diagnostic markers of CKD in the context of NAFLD, and it is important to note that CKD has the opposite effect on NAFLD, which is beyond the scope of our discussion; Third, our findings were required to validate *in vivo* and *in vitro* to better guide clinical practice, although the decreased expression of DUSPI and ZFP36 in CKD has been confirmed by related studies (36, 53).

## 5 Conclusion

In this study, 4 NAFLD-related genes (DUSP1, NR4A1, FOSB, ZFP36) were identified as diagnostic markers in CKD patients, and NAFLD may accelerate the development of CKD through immune and inflammatory pathways. The changes in immune cell infiltration in CKD and the significant correlation with diagnostic markers were also elucidated. Our study may provide diagnostic markers and therapeutic targets for CKD patients with NAFLD.

## Data availability statement

The datasets presented in this study can be found in online repositories. The names of the repository/repositories and accession number(s) can be found in the article/**Supplementary Material**.

## Author contributions

YC, YD, and WJ contributed equally to this work. YC and YD wrote the manuscript. WJ performed the data processing. JD, JY and HZ Participated in chart making. XZ edited the article. KT and ZY conceived and designed the scientific question. All authors contributed to the article and approved the submitted version.

## Supplementary material

Supplementary Materials S1 A list of 45 NAFLD-related shared genes.

Supplementary Materials S2 Results of GO and KEGG enrichment analysis of 45 NAFLD-related share genes.

Supplementary Materials S3 4 NAFLD-related hub genes were downregulated in GSE104954 dataset.

Supplementary Figure 1 Diagnostic value of 4 NAFLD-related hub genes in GSE104948 tested by ROC curves.

Supplementary Figure 2 Diagnostic value of 4 NAFLD-related hub genes in different sample sites of GSE32591 examined by ROC curves.

## References

[1] Stevens PE Levin A. Evaluation and management of chronic kidney disease: Synopsis of the kidney disease: Improving global outcomes 2012 clinical practice guideline. Ann Intern Med (2013) 158(11):825–30. doi: 10.7326/0003-4819-158-11-201306040-00007 23732715

[2] Liyanage T Toyama T Hockham C Ninomiya T Perkovic V Woodward M. Prevalence of chronic kidney disease in Asia: A systematic review and analysis. BMJ Glob Health (2022) 7(1):e007525. doi: 10.1136/bmjgh-2021-007525 PMC8796212 35078812

[3] Lin MY Fiorentino M Wu IW. Editorial: Public health for chronic kidney disease prevention and care. Front Public Health (2022) 10:1021075. doi: 10.3389/fpubh.2022.1021075 36176535 PMC9514552

[4] Jonsson AJ Lund SH Eriksen BO Palsson R Indridason OS. Incidence of and risk factors of chronic kidney disease: Results of a nationwide study in iceland. Clin Kidney J (2022) 15(7):1290–9. doi: 10.1093/ckj/sfac051 PMC9217641 35756731

[5] Tao Z Li Y Cheng B Zhou T Gao Y. Influence of nonalcoholic fatty liver disease on the occurrence and severity of chronic kidney disease. J Clin Transl Hepatol (2022) 10(1):164–73. doi: 10.14218/JCTH.2021.00171 PMC8845149 35233386

[6] Mantovani A Petracca G Beatrice G Csermely A Lonardo A Schattenberg JM. Non-alcoholic fatty liver disease and risk of incident chronic kidney disease: An updated meta-analysis. Gut (2022) 71(1):156–62. doi: 10.1136/gutjnl-2020-323082 33303564

[7] Yi M Peng W Feng X Teng F Tang Y Kong Q. Extrahepatic morbidities and mortality of NAFLD: An umbrella review of meta-analyses. Aliment Pharmacol Ther (2022) 56(7):1119–30. doi: 10.1111/apt.17165 35989292

[8] Byrne CD Targher G. NAFLD as a driver of chronic kidney disease. J Hepatol (2020) 72(4):785–801. doi: 10.1016/j.jhep.2020.01.013 32059982

[9] Powell EE Wong VW Rinella M. Non-alcoholic fatty liver disease. Lancet (2021) 397(10290):2212–24. doi: 10.1016/S0140-6736(20)32511-3 33894145

[10] Wang XJ Malhi H. Nonalcoholic fatty liver disease. Ann Intern Med (2018) 169(9):ITC65–80. doi: 10.7326/AITC201811060 30398639

[11] Musso G Gambino R Tabibian JH Ekstedt M Kechagias S Hamaguchi M. Association of non-alcoholic fatty liver disease with chronic kidney disease: A systematic review and meta-analysis. PloS Med (2014) 11(7):e1001680. doi: 10.1371/journal.pmed.1001680 25050550 PMC4106719

[12] Targher G Bertolini L Rodella S Lippi G Zoppini G Chonchol M. Relationship between kidney function and liver histology in subjects with nonalcoholic steatohepatitis. Clin J Am Soc Nephrol (2010) 5(12):2166–71. doi: 10.2215/CJN.05050610 PMC2994076 20724519

[13] Targher G Chonchol M Bertolini L Rodella S Zenari L Lippi G. Increased risk of CKD among type 2 diabetics with nonalcoholic fatty liver disease. J Am Soc Nephrol (2008) 19(8):1564–70. doi: 10.1681/ASN.2007101155 PMC2488256 18385424

[14] Park H Dawwas GK Liu X Nguyen MH. Nonalcoholic fatty liver disease increases risk of incident advanced chronic kidney disease: A propensity-matched cohort study. J Intern Med (2019) 286(6):711–22. doi: 10.1111/joim.12964 PMC6851415 31359543

[15] Sinn DH Kang D Jang HR Gu S Cho SJ Paik SW. Development of chronic kidney disease in patients with non-alcoholic fatty live disease: A cohort study. J Hepatol (2017) 67(6):1274–80. doi: 10.1016/j.jhep.2017.08.024 28870674

[16] Nassir F. NAFLD: Mechanisms, treatments, and biomarkers. Biomolecules (2022) 12(6):824. doi: 10.3390/biom12060824 35740949 PMC9221336

[17] Czaja-Stolc S Potrykus M Stankiewicz M Kaska Ł Małgorzewicz S. Pro-inflammatory profile of adipokines in obesity contributes to pathogenesis, nutritional disorders, and cardiovascular risk in chronic kidney disease. Nutrients (2022) 14(7):1457. doi: 10.3390/nu14071457 35406070 PMC9002635

[18] Grayson PC Eddy S Taroni JN Lightfoot YL Mariani L Parikh H. Metabolic pathways and immunometabolism in rare kidney diseases. Ann Rheum Dis (2018) 77(8):1226–33. doi: 10.1136/annrheumdis-2017-212935 PMC6045442 29724730

[19] Berthier CC Bethunaickan R Gonzalez-Rivera T Nair V Ramanujam M Zhang W. Cross-species transcriptional network analysis defines shared inflammatory responses in murine and human lupus nephritis. J Immunol (2012) 189(2):988–1001. doi: 10.4049/jimmunol.1103031 22723521 PMC3392438

[20] Nakagawa S Nishihara K Miyata H Shinke H Tomita E Kajiwara M. Molecular markers of tubulointerstitial fibrosis and tubular cell damage in patients with chronic kidney disease. PloS One (2015) 10(8):e0136994. doi: 10.1371/journal.pone.0136994 26317775 PMC4552842

[21] Arendt BM Comelli EM Ma DW Lou W Teterina A Kim T. Altered hepatic gene expression in nonalcoholic fatty liver disease is associated with lower hepatic n-3 and n-6 polyunsaturated fatty acids. Hepatology (2015) 61(5):1565–78. doi: 10.1002/hep.27695 25581263

[22] Bu D Luo H Huo P Wang Z Zhang S He Z. KOBAS-i: Intelligent prioritization and exploratory visualization of biological functions for gene enrichment analysis. Nucleic Acids Res (2021) 49(W1):W317–25. doi: 10.1093/nar/gkab447 PMC8265193 34086934

[23] Chai C Cox B Yaish D Gross D Rosenberg N Amblard F. Agonist of RORA attenuates nonalcoholic fatty liver progression in mice via up-regulation of MicroRNA 122. Gastroenterology (2020) 159(3):999–1014.e9. doi: 10.1053/j.gastro.2020.05.056 32450149 PMC7722250

[24] Chen L Chen L Li X Qin L Zhu Y Zhang Q. Transcriptomic profiling of hepatic tissues for drug metabolism genes in nonalcoholic fatty liver disease: A study of human and animals. Front Endocrinol (Lausanne) (2022) 13:1034494. doi: 10.3389/fendo.2022.1034494 36686439 PMC9845619

[25] Zeng T Chen G Qiao X Chen H Sun L Ma Q. NUSAP1 could be a potential target for preventing NAFLD progression to liver cancer. Front Pharmacol (2022) 13:823140. doi: 10.3389/fphar.2022.823140 35431924 PMC9010788

[26] Lonardo A Mantovani A Targher G Baffy G. Nonalcoholic fatty liver disease and chronic kidney disease: Epidemiology, pathogenesis, and clinical and research implications. Int J Mol Sci (2022) 23(21):13320. doi: 10.3390/ijms232113320 36362108 PMC9654863

[27] Chen HF Chuang HC Tan TH. Regulation of dual-specificity phosphatase (DUSP) ubiquitination and protein stability. Int J Mol Sci (2019) 20(11):2668. doi: 10.3390/ijms20112668 31151270 PMC6600639

[28] Kyriakis JM Avruch J. Mammalian MAPK signal transduction pathways activated by stress and inflammation: A 10-year update. Physiol Rev (2012) 92(2):689–737. doi: 10.1152/physrev.00028.2011 22535895

[29] Lu C Wu B Liao Z Xue M Zou Z Feng J. DUSP1 overexpression attenuates renal tubular mitochondrial dysfunction by restoring parkin-mediated mitophagy in diabetic nephropathy. Biochem Biophys Res Commun (2021) 559:141–7. doi: 10.1016/j.bbrc.2021.04.032 33940385

[30] Sheng J Li H Dai Q Lu C Xu M Zhang J. DUSP1 recuses diabetic nephropathy via repressing JNK-mff-mitochondrial fission pathways. J Cell Physiol (2019) 234(3):3043–57. doi: 10.1002/jcp.27124 30191967

[31] Ge Y Wu D Zhou Y Qiu S Chen J. lncRNA NR_038323 suppresses renal fibrosis in diabetic nephropathy by targeting the miR-324-3p/DUSP1 axis. Mol Ther Nucleic Acids (2019) 17:741–53. doi: 10.1016/j.omtn.2019.07.007 PMC6709345 31430717

[32] Al MHM Maniruzzaman M Shin J. Identification of key candidate genes for IgA nephropathy using machine learning and statisticsbased bioinformatics models. Sci Rep (2022) 12(1):13963. doi: 10.1038/s41598-022-18273-x 35978028 PMC9385868

[33] Liao Y Wang Z Wang L Lin Y Ye Z Zeng X. MicroRNA-27a-3p directly targets FosB to regulate cell proliferation, apoptosis, and inflammation responses in immunoglobulin a nephropathy. Biochem Biophys Res Commun (2020) 529(4):1124–30. doi: 10.1016/j.bbrc.2020.06.115 32819575

[34] Makita S Takatori H Nakajima H. Post-transcriptional regulation of immune responses and inflammatory diseases by RNA-binding ZFP36 family proteins. Front Immunol (2021) 12:711633. doi: 10.3389/fimmu.2021.711633 34276705 PMC8282349

[35] Ma J Li C Liu T Zhang L Wen X Liu X. Identification of markers for diagnosis and treatment of diabetic kidney disease based on the ferroptosis and immune. Oxid Med Cell Longev 2022 (2022) p:9957172. doi: 10.1155/2022/9957172 PMC9712001 36466094

[36] Zhou Y Yu Z Liu L Wei L Zhao L Huang L. Construction and evaluation of an integrated predictive model for chronic kidney disease based on the random forest and artificial neural network approaches. Biochem Biophys Res Commun (2022) 603:21–8. doi: 10.1016/j.bbrc.2022.02.099 35276459

[37] Safe S Jin UH Morpurgo B Abudayyeh A Singh M Tjalkens RB. Nuclear receptor 4A (NR4A) family - orphans no more. J Steroid Biochem Mol Biol (2016) 157:48–60. doi: 10.1016/j.jsbmb.2015.04.016 25917081 PMC4618773

[38] Ma G Chen F Liu Y Zheng L Jiang X Tian H. Nur77 ameliorates age-related renal tubulointerstitial fibrosis by suppressing the TGF-β/Smads signaling pathway. FASEB J (2022) 36(2):e22124. doi: 10.1096/fj.202101332R 34972249

[39] He Z Zhang M Xu H Zhou W Xu C Wang Z. Yiqi huoxue tongluo recipe regulates NR4A1 to improve renal mitochondrial function in unilateral ureteral obstruction (UUO) rats. Pharm Biol (2022) 60(1):2308–18. doi: 10.1080/13880209.2022.2148168 PMC9704077 36428248

[40] Westbrook L Johnson AC Regner KR Williams JM Mattson DL Kyle PB. Genetic susceptibility and loss of Nr4a1 enhances macrophage-mediated renal injury in CKD. J Am Soc Nephrol (2014) 25(11):2499–510. doi: 10.1681/ASN.2013070786 PMC4214519 24722447

[41] Chen J Huang XR Yang F Yiu WH Yu X Tang S. Single-cell RNA sequencing identified novel Nr4a1(+) Ear2(+) anti-inflammatory macrophage phenotype under myeloid-TLR4 dependent regulation in anti-glomerular basement membrane (GBM) crescentic glomerulonephritis (cGN). Adv Sci (Weinh) (2022) 9(18):e2200668. doi: 10.1002/advs.202200668 35484716 PMC9218767

[42] Tang PC Chan AS Zhang CB García CC Zhang YY To KF. TGF-β1 signaling: Immune dynamics of chronic kidney diseases. Front Med (Lausanne) (2021) 8:628519. doi: 10.3389/fmed.2021.628519 33718407 PMC7948440

[43] Lee H Fessler MB Qu P Heymann J Kopp JB. Macrophage polarization in innate immune responses contributing to pathogenesis of chronic kidney disease. BMC Nephrol (2020) 21(1):270. doi: 10.1186/s12882-020-01921-7 32660446 PMC7358194

[44] Han Y Ma FY Tesch GH Manthey CL Nikolic-Paterson DJ. Role of macrophages in the fibrotic phase of rat crescentic glomerulonephritis. Am J Physiol Renal Physiol (2013) 304(8):F1043–53. doi: 10.1152/ajprenal.00389.2012 23408165

[45] Anders HJ Ryu M. Renal microenvironments and macrophage phenotypes determine progression or resolution of renal inflammation and fibrosis. Kidney Int (2011) 80(9):915–25. doi: 10.1038/ki.2011.217 21814171

[46] Zhu X Li S Zhang Q Zhu D Xu Y Zhang P. Correlation of increased Th17/Treg cell ratio with endoplasmic reticulum stress in chronic kidney disease. Med (Baltimore) (2018) 97(20):e10748. doi: 10.1097/MD.0000000000010748 PMC5976317 29768353

[47] Bendickova K Fric J. Roles of IL-2 in bridging adaptive and innate immunity, and as a tool for cellular immunotherapy. J Leukoc Biol (2020) 108(1):427–37. doi: 10.1002/JLB.5MIR0420-055R PMC7384134 32480431

[48] Han Z Ma K Tao H Liu H Zhang J Sai X. A deep insight into regulatory T cell metabolism in renal disease: Facts and perspectives. Front Immunol (2022) 13:826732. doi: 10.3389/fimmu.2022.826732 35251009 PMC8892604

[49] So B Yap D Chan TM. B cells in primary membranous nephropathy: Escape from immune tolerance and implications for patient management. Int J Mol Sci (2021) 22(24):13560. doi: 10.3390/ijms222413560 34948358 PMC8708506

[50] Lafayette RA Canetta PA Rovin BH Appel GB Novak J Nath KA. A randomized, controlled trial of rituximab in IgA nephropathy with proteinuria and renal dysfunction. J Am Soc Nephrol (2017) 28(4):1306–13. doi: 10.1681/ASN.2016060640 PMC5373458 27821627

[51] Hartinger JM Kratky V Hruskova Z Slanar O Tesar V. Implications of rituximab pharmacokinetic and pharmacodynamic alterations in various immune-mediated glomerulopathies and potential anti-CD20 therapy alternatives. Front Immunol (2022) 13:1024068. doi: 10.3389/fimmu.2022.1024068 36420256 PMC9676507

[52] Owens EP Vesey DA Kassianos AJ Healy H Hoy WE Gobe GC. Biomarkers and the role of mast cells as facilitators of inflammation an fibrosis in chronic kidney disease. Transl Androl Urol (2019) 8(Suppl 2):S175–83. doi: 10.21037/tau.2018.11.03 PMC6559929 31236335

[53] Fu S Cheng Y Wang X Huang J Su S Wu H. Identification of diagnostic gene biomarkers and immune infiltration in patients with diabetic kidney disease using machine learning strategies and bioinformatic analysis. Front Med (Lausanne) (2022) 9:918657. doi: 10.3389/fmed.2022.918657 36250071 PMC9556813

