# Supplementary figures and images for: Identification of biomarkers for the diagnosis of chronic kidney disease (CKD) with non-alcoholic fatty liver disease (NAFLD) by bioinformatics analysis and machine learning
Source: Front Endocrinol (Lausanne). 2023 Feb 27;14:1125829. doi: 10.3389/fendo.2023.1125829 (PMC10009268; doi:10.3389/fendo.2023.1125829)

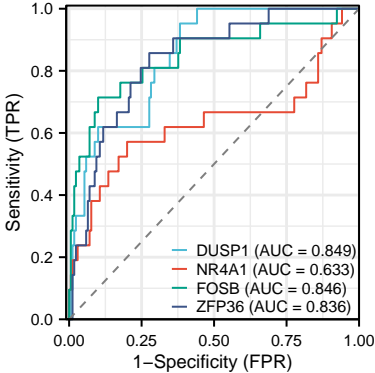

Supplement: Supplementary Figure 1 — Diagnostic value of 4 NAFLD-related hub genes in GSE104948 tested by ROC curves. [file DataSheet_1.pdf]

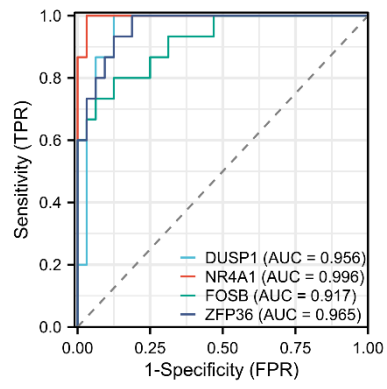

Tubulointerstitial

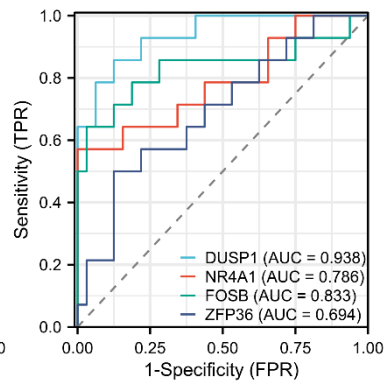

Glomerular

Supplement: Supplementary Figure 2 — Diagnostic value of 4 NAFLD-related hub genes in different sample sites of GSE32591 examined by ROC curves. [file DataSheet_2.pdf]
